# A systematic assessment of the stability of SLA® vs. SLActive® implant surfaces over 12 weeks

**DOI:** 10.1038/s41432-024-01097-1

**Published:** 2025-01-07

**Authors:** Rohit Patel, Serena Patel, Wail Girgis, Waqar Ahmed, Fadi Barrak

**Affiliations:** 1 School of Medicine and Dentistry, University of Central Lancashire, Preston, UK; 2 Downing College, University of Cambridge, Cambridge, UK; 3 Omnia Academy, Omnia, UK; 4 Devonshire House, Cambridge, UK; 5 School of Mathematics and Physics, University of Lincoln, Lincoln, UK; 6 VSS Academy, London, UK

**Keywords:** Dental materials, Dental treatments

## Abstract

**Objective:**

This study aims to assess the impact of two implant surfaces, SLA and SLActive, on implant stability, measured by ISQ levels over a 12-week period.

**Methods:**

A comprehensive search of MEDLINE, EMBASE, Cochrane Central Register of Controlled Trials, and Dentistry and Oral Sciences databases for randomized controlled trials (RCTs) up to February 2023 was conducted. The inclusion criteria were studies involving adult patients treated with SLA and SLActive implants, with assessment of implant stability through ISQ levels up to 12 weeks post-placement.

**Results:**

From the initial 180 potentially eligible publications identified, six RCTs were included in our analysis, comprising 326 implants (50.6% SLA and 49.4% SLActive). Three studies were classified as low risk, while three had an unclear risk of bias. Overall, SLActive implants demonstrated comparable stability levels, as measured by ISQ, to SLA implants within the 12-week interval for implants placed in the maxillary or mandibular region. However, findings from the RCTs suggest that the SLActive surface led to an earlier transition point, a faster return to stability levels, and higher ISQ values at the end of 12 weeks compared to the SLA surface for implants placed in the palatal region.

**Conclusion:**

SLActive surfaces exhibited stability levels similar to SLA surfaces for maxillary and mandibular implants. Notably, for palatal implants, SLActive resulted in a quicker transition point and higher stability levels at the 12-week mark. Due to the limited number of trials and potential study heterogeneity, further research is needed to validate these findings.

Key points

- Our systematic review addresses a critical gap in current literature by rigorously evaluating the implant stability of two prominent surfaces, SLA and SLActive, over a 12-week period. Implant stability is paramount in prosthodontics, directly impacting treatment success and patient satisfaction.
- The choice between SLA and SLActive surfaces is a daily consideration for prosthodontists, implantologists, and researchers alike. As *Evidence Based Dentistry* is dedicated to advancing clinical knowledge and practice, our review directly contributes to this mission by presenting evidence that can guide practitioners in selecting the most suitable implant surface for enhanced stability and long-term success. We have meticulously scrutinized the methodologies employed in studies comparing implant stability, ensuring that our review not only synthesizes existing data but also provides a critical evaluation of the research landscape. This methodological focus aligns with the journal's commitment to promoting evidence-based practices in prosthodontics.
- As *Evidence Based Dentistry* has a keen interest in patient-centered care, our research explores not only the quantitative aspects of implant stability but also delves into the qualitative implications for prosthodontic interventions. This holistic approach provides a comprehensive view that goes beyond numerical data, offering valuable insights into the practical implications for patient care. Our manuscript explores the implications of implant surface characteristics on long-term patient outcomes.
- This focus adds a dimension to our research that is directly relevant to the overarching goals of the journal. In addition, the timing of our study is particularly relevant, given the dynamic landscape of prosthodontics and the continuous evolution of implant technologies. By submitting this paper to *Evidence Based Dentistry*, we aim to contribute to the ongoing discourse within the field, assisting clinicians and researchers in staying abreast of the latest advancements and evidence-based practices.

## Introduction

Dental implants play a crucial role in replacing missing teeth, emphasizing the importance of achieving both primary and secondary stability. Osseointegration, the essential connection between the implant and living bone, is a key determinant for long-term success. Osseointegration is evaluated through bone-to-implant contact (BIC) under a microscope, without a specific minimum requirement^1–5^.

The osseointegration process begins with primary mechanical stability, transitioning gradually to secondary biological stability through the dynamic balance of bone resorption and formation. Initially, the implant establishes contact with surrounding bone, providing primary stability. Over time, osteoclasts resorb this bone, replaced by newly formed viable bone and bone growth on the implant surface, leading to secondary stability.

Early implant loss risks are heightened when osseointegration falters due to movement between the implant and bone during the early healing phase, typically between two to three weeks post-surgery. The attainment of sufficient stability depends on factors such as implant design (both micro-design and macro-design) which influence the shift from primary mechanical stability to secondary stability through osseointegration.

Surface modification of dental implants is crucial to enhancing osseointegration and reducing healing time. This involves creating microrough titanium surfaces like SLA, which improves bone-implant contact. Surface energy, influencing hydrophilicity and promoting osteogenesis, plays a role in this process.

Both SLA and SLActive surfaces are composed of coldworked titanium, sandblasted with large grit corundum, and acid-etched with sulfuric and hydrochloric acid^1,4^. However, SLActive implants, unlike SLA implants, undergo rinsing under protective nitrogen gas conditions, preventing air exposure and storing in a sealed tube with isotonic NaCl solution. This unique process imparts SLActive implants with higher surface energy and greater hydrophilicity than SLA implants^2,3^. These modifications facilitate stronger cell reactions and bone tissue response, accelerating early bone deposition, reducing healing time, and improving patient quality of life while decreasing implant failure. Studies show that SLActive surfaces promote greater osseointegration compared to SLA surfaces in both animal and human studies^6,7^.

While few studies have directly compared implant stability between SLA and SLActive implants in humans, most research relies on animal and in vitro studies^6–8^. Animal experiments have indicated faster osseointegration with SLActive implants within four weeks compared to SLA implants. Clinically, measuring implant stability directly is challenging, but indirect methods like the implant stability quotient (ISQ) are commonly used, where higher ISQ values suggest better osseointegration^8^.

ISQ levels, typically on a scale from 1 to 100, with values between 55 and 80 considered optimal for implant success, initially decrease in the early healing phase due to reduced primary stability, followed by an increase indicating the transition from bone resorption to formation, resulting in secondary stability. The surface modification of SLA and its impact on ISQ levels and the transition from primary to secondary stability are crucial, especially for early or immediate implant loading^8^.

Patient non-compliance factors, including implant overloading and oral hygiene, can influence the healing process and increase the risk of implant failure. Despite a lack of systematic reviews and limited studies in this area, this systematic review aims to determine whether SLActive implants improve implant stability compared to SLA implants. The goal is to provide valuable insights for optimized loading protocols and informed implant selection, ultimately leading to better long-term outcomes for patients.

This review aims to determine whether the SLActive surface enhances implant stability, as indicated by ISQ levels, in comparison to the SLA implant surface over a 12-week period^5^. For individuals undergoing dental implant treatment, does the use of SLActive surface implants lead to improved implant stability, measured by ISQ levels within intervals up to 12 weeks, compared to the use of SLA surface implants?

PICO Terms Identified: Population - Individuals undergoing dental implant treatment for the replacement of single or multiple maxillary and mandibular teeth. Intervention – The utilization of SLActive surface implants. Comparison - The utilization of SLA surface implants. Outcome - Assessment of implant stability through ISQ levels within intervals up to 12 weeks.

## Materials and methods

A thorough search across OVID MEDLINE, OVID EMBASE, the Cochrane Central Register of Controlled Trials, and Dentistry & Oral Sciences databases to identify relevant studies up to February 2023 was conducted. Utilizing existing study design filters, we applied filters to limit results to human studies. Our searches combined controlled vocabulary terms with text word searches, and we undertook searches without restrictions on publication dates or language to ensure a comprehensive literature search. The query focused on MeSH terms and keywords reflecting our research objectives: (1) Implant stability, (2) SLA, and (3) SLActive. Inclusion and exclusion criteria are detailed in Table 1.

**Table 1:** Inclusion & exclusion criteria.

	Inclusion	Exclusion
**Types of participants**	Adult patients having implant treatment to replace one or more maxillary / mandibular teeth Adult patients who were having orthodontic treatment with palatal implants (as temporary anchorage devices)	Animal studies Paediatric studies
**Types of interventions**	SLActive surface modified implants to replace one or more maxillary or mandibular teeth or those undergoing orthodontic treatment with SLActive palatal implants.	
**Types of outcome measure**	ISQ levels at regular intervals up to 12 weeks post-placement	Studies measuring marginal bone loss as the only outcome measure Studies that are less than 12 weeks long
**Types of studies**	Randomized controlled trials	Studies with no control of comparator Retrospective studies Animal studies Cross-over studies Case-control studies In vitro studies

**Fig. 1:**
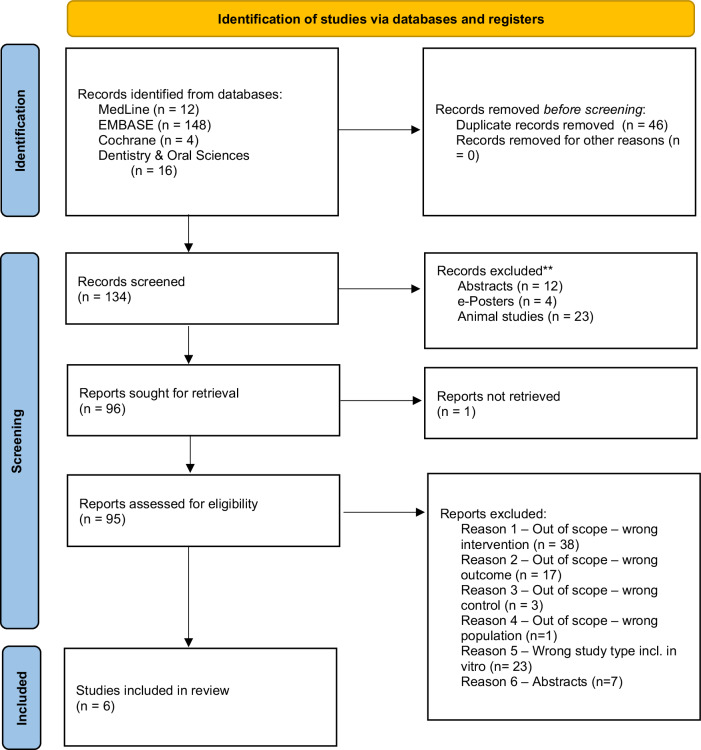
Preferred reporting items for systematic reviews and meta-analyses (PRISMA) flow chart.

Each database was searched individually, exporting results to RefWorks for de-duplication. Publication titles were screened for eligibility, with appropriate ones retained. Abstracts of the remaining articles were read in full, and suitable articles were further assessed. Full-text articles meeting inclusion and exclusion criteria were retained for the review. We employed a PRISMA flow chart (Fig. 1) to summarize the study selection process.

Relevant data was extracted from the selected studies and presented it in a tabulated form. This included study type, authors, sponsors, trial ID if applicable, year of publication, inclusion/exclusion criteria, participant details, detailed description of interventions and comparators, outcomes reported, assessment methods, and details of blinding, sample size calculations, funding, and conflicts of interests.

**Fig. 2:**
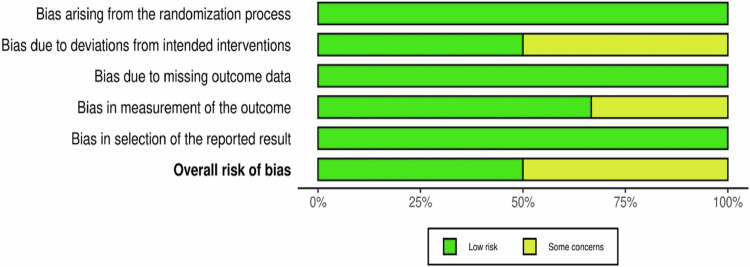
'Risk of Bias' graph: judgment about each 'Risk of Bias' domain, presented as percentages across all included studies.

**Fig. 3:**
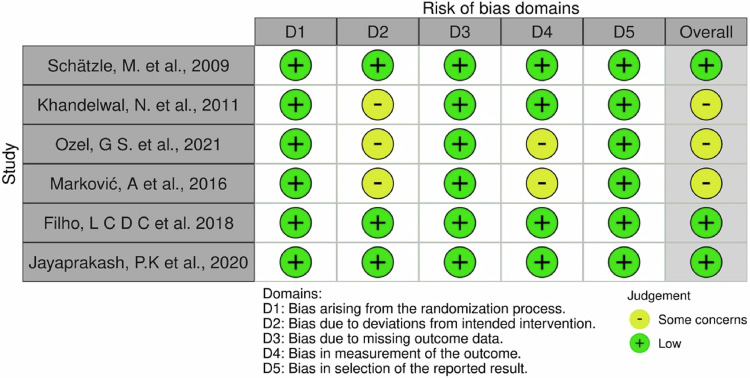
'Risk of Bias' domains: judgment about each 'Risk of Bias' domains.

The Cochrane Risk of Bias 2 (RoB 2) tool was used for a comprehensive risk of bias assessment^9,10^. Individual studies were categorized as low, high, or having some concerns raised regarding risk of bias (Figs. 2 and 3). Factors such as plausible bias, the likelihood of altering results, and overall study quality were assessed. The assessment results were graphically presented using Robvis software.

The results were presented through a narrative descriptive synthesis, providing ISQ values as mean ± Standard Deviation.

Ethical clearance for this review was obtained from an anonymized university institution.

## Results

A total of 180 potentially eligible records were initially identified from the databases. After removing duplicates, 134 records were screened, and 96 reports were sought for retrieval. Subsequently, 95 records were assessed for eligibility, resulting in the inclusion of six Randomized Controlled Trials (RCTs)^11–16^ in this systematic review. The PRISMA diagram in Fig. 1 illustrates the total number of papers identified and excluded at each stage.

Across the six RCTs, a total of 155 patients and 326 implants were included, comprising 165 (50.6%) control SLA implants and 161 (49.4%) test SLActive implants. The main characteristics and outcomes are summarized in Supplementary Table 1. In three studies^11,12,15^, ISQ levels in SLA and SLActive implants showed a similar trend of initial decrease followed by an increase until the study endpoint. However, two studies^11,15^ reported an immediate increase in ISQ levels after implant placement, followed by a decrease and subsequent increase, with similar trends observed for both SLA and SLActive implants.

For implants in the palatal region^11,15^, SLActive demonstrated a significantly earlier transition point at 4 weeks compared to 5 weeks for SLA. While one study^14^ indicated an ISQ minimum at three weeks for SLActive implants compared to four weeks for SLA, the difference was not significant. Other RCTs showed no significant difference in the transition point between SLA and SLActive groups^12,13,16^.

Three RCTs^12,13,16^ found no significant difference in the trends of ISQ values between SLA and SLActive throughout the study. However, two studies^11,15^ reported that ISQ levels increased significantly more over time in the SLActive group compared to SLA, with SLActive reaching a minimum at 4 weeks compared to 5 weeks for SLA.

Considering ISQ values at 12 weeks, only the two palatal implant studies^11,15^ reported significantly higher implant stability with SLActive compared to SLA. Other RCTs^12–14,16^ noted no significant difference in ISQ levels at 12 weeks between SLActive and SLA surface implants.

All included studies^11–16^ were considered to be of low risk of bias regarding the randomization process. Some concerns regarding bias due to deviations from intended interventions were raised in Khandelwal et al.’s study^12^ but did not lead to exclusion. Similarly, Markovic et al. and Sayin Ozil et al.'s studies^13,16^ had some concerns about blinding, but they were not excluded as the concerns were not of high risk. The remaining studies^11,14,15^ were deemed to be of low risk of bias regarding deviations from intended interventions.

No studies had missing outcome data, and all were determined to be of low risk of bias in this domain. While methods of measuring the outcome were appropriate in all included studies^11–16^, masking of outcome assessors was confirmed in only four studies^11,12,14,15^, with some concerns raised in the remaining two^13,16^. Despite these concerns, the studies were not excluded, as the potential bias was unlikely to impact observer-reported outcomes.

All studies showed no bias in the selection of reported results. Overall, three studies were deemed to be of low risk of bias^11,14,15^, while three were considered to be at unclear risk of bias due to some concerns raised^12,13,16^. Figs. 2 and 3 provide a visual representation of the overall risk of bias for all included studies across all domains and for each individual study across each domain, respectively.

## Discussion

This systematic review scrutinized whether the SLActive implant surface contributes to enhanced implant stability measured by ISQ levels within a 12-week timeframe. Among the included studies, only two^14,15^ reported that SLActive surfaces led to an earlier transition point, higher ISQ levels, and a superior ISQ value at the end of the 12-week period compared to SLA. One study^14^ noted an earlier transition point but similar ISQ trends. In contrast, the remaining studies^12,13,16^ did not reveal significant differences between SLA and SLActive concerning transition points, ISQ trends, and implant stability measured by ISQ levels within the 12-week period. Overall, the studies suggest that both SLA and SLActive implant surfaces may yield comparable ISQ levels in the first 12 weeks following implant placement, with acceptable ISQ levels (>=70) observed in all included studies. Notably, the two studies reporting significant improvements with SLActive involved implants in the mid-palatal region^11,15^, differing from anterior/posterior mandibular or maxillary jaw placements. Caution is warranted when comparing these studies due to variations in implant types and loading protocols. The trends in ISQ levels typically showed an initial decline, reflecting bone resorption, followed by an increase, signifying new bone formation and osseointegration. One study^13^ didn't reach baseline stability by 12 weeks, possibly due to patient conditions (anticoagulant use) affecting ISQ levels.

This systematic review assessed implant stability based on ISQ levels within 12 weeks. Three of the included studies had an unclear risk of bias, while the remaining three were deemed low risk. Critically appraising trial quality is crucial for detecting flaws and ensuring transparency in methodology. However, the review couldn't perform a meta-analysis due to slight heterogeneity in reported test and control groups. Multiple implants in some patients and insufficiently detailed data hindered the pooling of estimates, considering potential confounding factors. Study designs varied, leading to potential biases with pooled estimates. Various factors, including comorbidities, different implant sites, varied implant designs, lengths, diameters, and restorative protocols, posed limitations. The site of implant insertion impacts osseointegration and the risk of implant failure. Studies didn't uniformly record bone quality assessments, influencing implant stability. Variations in implant types (parallel-walled vs. tapered), bone-level vs. tissue-level implants, implant length, and platform diameter further complicated comparisons. Differences in post-procedural care, such as antibiotic choice, duration, and advice, may have influenced implant stability, and the choice of implant material and its effects on stability wasn't consistently documented.

In summary, a multitude of factors, including patient conditions, implant site, design, and materials, made it challenging to draw meaningful and direct comparisons in this review. Literature comparing SLA and SLActive surface implants presents variability. Animal and human studies have suggested potential benefits of SLActive implants over SLA implants. Animal studies demonstrated faster bone integration with SLActive implants, while human histological studies indicated greater bone-to-implant contact in the early weeks, diminishing after six weeks.

In human clinical studies, ISQ levels were similar after six weeks for SLA and SLActive implants, with variations in transition points. Most studies in this systematic review found no significant ISQ differences after 12 weeks. However, some studies reported improved implant stability with SLActive implants, while others showed negligible differences in longer-term outcomes. Research on SLA and SLActive implants has yielded mixed results concerning implant stability, with varying outcomes across different studies.

## Conclusions

The review suggests that SLActive implants demonstrate comparable implant stability to SLA implants within the initial 12 weeks when placed in the maxillary or mandibular region. However, for palatal implants, SLActive surfaces may lead to an earlier transition point, a quicker return to baseline stability, and higher ISQ values at the end of 12 weeks compared to SLA surfaces. This indicates that palatal implants with SLActive surfaces may achieve secondary stability faster and could potentially be loaded earlier.

The limitations of this review, including the limited number of available randomized controlled trials (RCTs), study heterogeneity, and the absence of pooled estimates, present significant challenges in drawing definitive conclusions. To gain a more comprehensive understanding of the impact of SLA vs. SLActive implants on osseointegration and implant stability, further studies are crucial. These studies should take into account various factors such as the implant placement site, bone quality, and potential effects of medical conditions and medications. Moreover, future research could explore the application of SLActive implants in orthodontics as temporary anchorage devices, particularly in palatal locations, to investigate the potential for earlier or increased loading.

## Supplementary information

PRISMA Checklist

SI Table 1

## Data Availability

The data that supports the findings of this study are available in the supplementary material of this article.
