# Supplementary material for: A systematic assessment of the stability of SLA® vs. SLActive® implant surfaces over 12 weeks
Source: Evid Based Dent. 2025 Jan 7;26(1):67–8. doi: 10.1038/s41432-024-01097-1 (PMC11953047; doi:10.1038/s41432-024-01097-1)
Supplement: Supplementary file 2 — SI Table 1 [file 41432_2024_1097_MOESM2_ESM.pdf]

Supplementary Table 1: Study Characteristics Table

Study	Methods	Participants & Procedures	Outcome measures	Main Results	Conclusions	Notes
Schätzle *et al.*, 2009	RCT with parallel groups	40 patients<br><br>Systemically well<br><br>19F 21M<br><br>21.3 – 51.8years<br><br>Midpalatal maxilla<br><br>Control group: 20 SLA implants<br><br>Test group: 20 SLActive implants	Clinical evaluation: ISQ difference from baseline, time-dependent stability patterns for each implant type to identify transition point	Transition point: SLActive at 28 days, SLA control group at 35 days<br><br>Reached baseline value: SLActive by 40 days, SLA by 63 days<br><br>End point ISQ value: higher for SLActive compared to SLA (77.8±1.9 vs 74.5±3.977)	Transition point achieved 1 week earlier (4 weeks vs 5 weeks) with SLAactive compared to SLA<br><br>ISQ level was greater for SLActive at transition point.<br><br>At 12 weeks ISQ level was statistically greater for SLActive compared to SLA<br><br>SLActive reached baseline value quicker (40 vs 63 days)	Zurich, Switzerland

Khandelwal *et al.*, 2013	RCT with split-mouth design	24 patients<br><br>Adults with a diagnosis of Type 2 diabetes missing at least two posterior mandibular teeth<br><br>15F 9M<br><br>38 – 76years<br><br>Posterior mandible<br><br>Control group: 24 SLA implants	Clinical evaluation: baseline ISQ, the minimum ISQ, the time following implant placement at which the minimum ISQ was observed, the ISQ at 16 weeks, and the time to healing (the first time after 8 weeks at which the ISQ was equal to or greater than baseline ISQ). Analysed using ANOVA	There were neither significant main effects of implant type (P > 0.6565) nor were there any significant interactions of implant type with time following implant placement (P > 0.3140).<br><br>Transition point: 3.5 ± 0.3 (SLActive) vs 4.2 ± 0.4 (SLA) though difference is non-significant<br><br>ISQ levels declined after implant placement, and by week 8 reached baseline measurements; showed increases in ISQ levels through weeks 10–12 and then changed minimally from week 12 to 16.	Similar ISQ levels throughout study<br><br>Reached baseline at 8 weeks, increased 10-12 weeks then minimal change up to 16 weeks<br><br>Transition point: 3.5 ± 0.3 (SLActive) vs 4.2 ± 0.4 (SLA) though difference is non-significant	University of Texas Health Science Center at San Antonio (UTHSCSA) Dental School

		Test group: 24 SLActive implants				
Sayin Ozel *et al.*, 2021	RCT with split-mouth design	12 patients<br><br>Systemically well<br><br>20- 50years<br><br>Mandible<br><br>Control group: 25 SLA implants<br><br>Test group: 25 SLActive implants	Clinical evaluation: ISQ levels at baseline then weekly for the next six weeks then monthly for the next 3 months. Data analysed with ANOVA	No significant differences were observed between the groups.<br><br>For both implant surfaces, an increase was observed over time, with no significant differences between the groups.<br><br>There were no significant differences between<br><br>SLA and SLActive surfaces in terms of stability ($P > 0.05$)	Similar ISQ levels throughout study<br><br>Exceeded baseline at all points following baseline<br><br>No significant difference between ISQ levels at 12 weeks	Selcuk University, Istanbul, Turkey

				Exceeded baseline at all points following placement<br><br>Same mean at 12 weeks ISQ (81.67 ± 0.33 vs 81.67 ± 0.33)		
Marković *et al.*, 2017	RCT with split-mouth design	20 patients<br><br>Receiving anticoagulants with INR between 1.2-2.5<br><br>4F 16M<br><br>59-69years<br><br>Anterior maxilla (25), posterior maxilla (17),	Clinical evaluation: ISQ levels at baseline, 1-, 2, and 3-week and 1-, 2-, and 3-month intervals. Data analysed with ANOVA<br><br>The implant survival was defined as the implant being in place and stable in	No significant differences in ISQ values at any point between two groups (P = 0,968; test statistics F = 0,002)<br><br>Transition point at 3 weeks in both groups<br><br>Failed to reach baseline in both groups after 12 weeks, though increased steadily up to 3 months after placement and stabilised.	Similar ISQ levels throughout the study<br><br>Transition point at 3 weeks<br><br>Failed to reach baseline in both groups<br><br>End point ISQ similar in both groups	School of<br><br>Dental Medicine, University of Belgrade, Serbia, and University Catholica of San Antonio<br><br>de Murcia, Spain

		anterior mandible (10), posterior mandible (28)<br><br>Control group: 40 SLA implants<br><br>Test group: 40 SLActive implants	the time of assessment.			
Carmo Filho *et al.*, 2018	RCT with split-mouth design	19 patients<br><br>Systemically well adults with bilaterally edentulous mandibles<br><br>12F 7M	Clinical evaluation: trends in ISQ value from baseline to 91 days post-surgery including occurrence of the transition point. Analysed using ANOVA, paired t-test and Bonferroni test	Transition point: SLActive at 3weeks, SLA control group at 4 weeks<br><br>Exceeded baseline value throughout study<br><br>SLActive implants showed significantly lower ISQ values than SLA implants at 5 weeks ($p <$	Transition point achieved 2 weeks earlier (3 weeks vs 5 weeks) with SLAactive compared to SLA<br><br>Exceeded baseline value throughout study	São Leopoldo Mandic Dental Research Center<br><br>(Campinas / SP), Brazil

		Age range not given, mean 46.7<br><br>Mandible<br><br>Control group: 36 SLA implants<br><br>Test group: 32 SLActive implants		0.001) and this persisted to seven weeks, from the eighth week SLA and SLActive implants had similar ISQ levels, which steadily increased to 12 weeks post implant placement.<br><br>End point ISQ: 82 ± 2.2 (SLA) vs 81 ± 1.7 (SLActive) no significant difference	At 12 weeks ISQ level was showed no significant difference between SLActive and SLA	
Jayaprakash *et al.*, 2020	RCT with parallel groups	40 patients<br><br>Systemically well adults<br><br>24F 16M<br><br>22.3 – 54.8years Mean 26.9	Clinical evaluation: ISQ difference from baseline, time-dependent stability patterns for each implant type (Chow test) to identify transition point	Transition point: SLActive at 4 weeks, SLA control group at 5 weeks<br><br>Reached baseline value: SLActive by 6 weeks, SLA by 9 weeks	Transition point achieved 1 week earlier (4 weeks vs 5 weeks) with SLAactive compared to SLA<br><br>ISQ level was greater for SLActive at transition point.	Kothiwal Dental College and Research Center, Mora Mustaqueem, Moradabad, UP, India

		Midpalatal region of maxilla<br><br>Control group: 20 SLA implants<br><br>Test group: 20 SLActive implants		End point ISQ value: SLActive: 78.68 ± 2.9 compared with the SLA implants of 75.5 ± 3.19, respectively.	At 12 weeks ISQ level was statistically greater for SLActive compared to SLA	
